# Cumulative Prospect Theory: Performance Evaluation of Government Purchases of Home-Based Elderly-Care Services Using the Pythagorean 2-tuple Linguistic TODIM Method

**DOI:** 10.3390/ijerph17061939

**Published:** 2020-03-16

**Authors:** Jianping Lu, Tingting He, Guiwu Wei, Jiang Wu, Cun Wei

**Affiliations:** 1 School of Business, Sichuan Normal University, Chengdu 610101, China; lujp2002@163.com; 2 School of Statistics, Southwestern University of Finance and Economics, Chengdu 611130, China; wujiang@swufe.edu.cn (J.W.); weicun1990@163.com (C.W.)

**Keywords:** multiple attribute group decision making (MAGDM), Pythagorean 2-tuple linguistic sets (P2TLSs), TODIM method, cumulative prospect theory (CPT), performance evaluation, home-based elderly-care service

## Abstract

The aging trend of China’s population is increasing, and the pension problem is becoming increasingly prominent. The pension mode provided by the government alone can no longer meet the social demand, and the government’s purchase of home-based care services from social organizations has become a new trend. In order to improve the efficiency and quality of pension services, a reasonable performance evaluation model needs to be established. Performance evaluations of home-based elderly-care services purchased by the government are problematic as a result of multiple-attribute group decision-making (MAGDM), as the problems are not single-attribute or single-expert issues. The extended TODIM not only integrates the advantages of cumulative prospect theory (CPT) into a consideration of the psychological factors of DMs, but also retains the superiority of the classical TODIM in relative dominance. The Pythagorean 2-tuple linguistic sets (P2TLSs) could easily depict qualitative assessment information related to the government’s purchase of home-based care services. Thus, in this paper, we extend the TODIM method based on the cumulative prospect theory (CPT) to the Pythagorean 2-tuple linguistic sets (P2TLSs) and propose a Pythagorean 2-tuple linguistic CPT-TODIM (P2TL-CPT-TODIM) method for MAGDM. The P2TL-CPT-TODIM method was proven superior to the classical one through a case study that included a performance evaluation of a home-based elderly-care service purchased by the government. Meanwhile, a comparison with the P2TL-CPT-TODIM method was performed to demonstrate the stability and effectiveness of the designed method.

## 1. Introduction

Home-based elderly-care services that have been purchased by the government involve three parties, namely, the government, social organizations and the public. Home-based elderly-care services are purchased by the government from social organizations to meet the public demand. Thus, a canny government purchase will result in matching inputs and outputs and an optimal performance. The performance is used to measure the government pension service supply capacity by means of an accurate performance evaluation. However, at the current stage, the government purchasing-endowment service in China has few scholars for pension services through which to conduct a comprehensive and reasonable performance assessment. Drucker [1] points out that social innovation is of great value and a breakthrough in social management is needed. The method of purchasing services through public service agencies is a kind of social innovation, which creates the proper value for society. Najam [2] believes that government and social organizations collaborate through four modes: cooperative, enveloping, complementary and confrontational. The governmental purchase of old-age services fits one of the collaborative modes. Hastak [3] points out that the public acceptance of public services plays an important role in the public services themselves. Revilla et al. [4] introduced a DEA method to evaluate performances in public–private partnerships, and Berrios [5] considered that the validity of the results will ultimately affect assessment services. Ancarani [6] introduced the idea of public satisfaction when assessing quality of service, and used a Value Customer (VC) model to carry out the performance evaluation. In terms of the indicator system, Grizzle [7] believes that relevant government performance evaluations should consider many aspects including efficiency, quality, cost-effectiveness, fairness, consistency of policies, and stability of inputs. Kearney and Berman [8] have pointed out that public sector performance evaluations should be guided by many aspects such as fairness, efficiency, and effectiveness. Ammons [9] believes that performance evaluations should be diversified in terms of output, quality, efficiency, fairness, results, value, customers. Waters [10] assumed that the selection of performance evaluation indicators is related to and development level of a country. In more-developed countries, quality and results should be the focus of attention, while in less-developed countries more attention should be paid to the purchase environment and buyer qualifications. Huang [11] used a sample of the Dutch industry-wide pension fund and suggested that its Z-score should be used to report its investment performance.

Gomes and Lima [12] were the first to propose the traditional TODIM. Because of the complexity of the decision environment, each alternative needs to be considered from different respects, while also considering the scheme and its relative superiority to other schemes. Therefore, the TODIM method is an ideal multiple attribute decision making (MADM) method, although this approach has limitations. For instance, it does not require an appropriate method to determine the weights of attributes, and does not provide a comprehensive approach. For this reason, Tian et al. [13] improved the classic TODIM method and combined it with the cumulative prospect theory (CPT) to transform the weight of attributes, resulting in more realistic decisions. 

There are three reasons why the TODIM method was selected in this study as the most useful DM tool for dealing with performance evaluation issues of home-based elderly-care services that have been purchased by the government. The first reason is that the performance evaluation of DMs is complex and DMs are needed in order to depict evaluation information from various aspects, and the TODIM method is one of the most popular tools for performance evaluation issues. Secondly, investigating the superiority of evaluation information not only takes into account the advantages of the evaluation alternatives, but also considers the method’s relative superiority compared with other evaluation alternatives. The relative assessment of an evaluation alternative is precisely depicted by the TODIM method, and an overall dominance of one evaluation alternative compared to all the others is derived through the TODIM method. Most importantly, these investigations are made by DMs whose performance evaluation information may be more or less affected by their psychological states. Moreover, the TODIM method is built based on the CPT, which is an optional and effective method for reflecting the psychological behaviors of DMs. Thus, the TODIM based on the CPT is adopted in this study as the basic performance evaluation tool.

On the other hand, MAGDM is an interesting and complex day-to-day issue involving implicit uncertainty and vagueness. Intuitionistic fuzzy sets (IFSs) [14] are a powerful extension of fuzzy sets [15] that allow multiple degrees of truth to be associated with each information preference for a better depiction of uncertainty and vagueness. Pythagorean fuzzy sets (PFSs) [16] have appeared as the valid means to describe MADM issues with uncertain information. There are two depicted variable degrees in PFSs—membership and non-membership—whose sums of squares cannot exceed 1. Thus, PFSs are more general than IFSs. However, all the above approaches are unsuitable for depicting the membership and non-membership degrees of an element with a linguistic label (which can express the decision-maker’s confidence level when they are making an evaluation). In order to overcome this limitation, Wei et al. [17] proposed Pythagorean 2-tuple linguistic sets (P2TLSs) based on the PFSs [18] and 2-tuple linguistics [19]. Huang and Wei [20] used the TODIM method to solve MADM with P2TLSs. Tang and Wei [21] designed some generalized BM operators to solve the MADM issues for green supplier selection under P2TLSs. He et al. [22] developed the P2TL-VIKOR method for evaluating human factors in construction project management. T.T. He et al. [23] built the P2TL-Taxonomy method for supplier selection in medical instrument industries. Although these approaches can effectively be applied to solve the Pythagorean 2-tuple linguistic MADM or MAGDM issues, they do have some limitations: (1) all these methods do not consider the objective weight information; (2) Pythagorean 2-tuple linguistic aggregating operators, the P2TL-VIKOR method, and the P2TL-Taxonomy method do not consider the behavioral factors of DMs during the actual decision-making process; (3) the P2TL-TODIM method only considers the psychological states of DMs during the decision-making process, but the P2TL-TODIM method does not depict the irrational decision-making behavior of DMs under uncertainty, which actually represents a lot of irrational behavioral factors during the decision-making process.

Solving the problems of a performance evaluation of home-based elderly-care services purchased by the government depends on multiple-attribute group decision-making (MAGDM), as they are not a single- attribute or single-expert problem. In this respect, MAGDM techniques or tools can be used to investigate problems in a better way. MAGDM methods are used to express reasonable performance evaluations or to choose the most appropriate and favorable performance evaluation alternatives on the basis of multiple attributes and numerous experts. Thus, the performance evaluation problems of home-based elderly-care services purchased by the government can be regarded as classical MAGDM issues in which many experts consider multiple attributes. The most important reason to select P2TLSs as the most useful tool for DMs in dealing with the performance evaluation issues of home-based elderly-care services that have been purchased by the government in this study is that P2TLSs can effectively depict the imprecise or vague information during this kind of performance evaluation. Based on this, the main aim of this paper is to provide a method of performance evaluation for government home endowment services, improving the satisfaction of the service object and promoting the health and orderly development of China’s home endowment service society. In this paper, we extend this novel TODIM method based on the CPT to the P2TLSs and take the limited rationality of decision-maker thinking into a more comprehensive consideration, using the P2TLSs to convey experts’ evaluations of each alternative for each attribute. This combination has a prospective application in corresponding cases, and can boost and replenish the current research. It is interesting to apply this research topic to MAGDM in order to select the best alternative. Thus, the objective of this study is to evaluate the performance evaluation of home-based elderly-care services purchased by the government with P2TLSs. The innovations and contributions can be listed as follows: (1) the TODIM is extended along with cumulative prospect theory (CPT) under P2TLSs; (2) the Pythagorean 2-tuple linguistic CPT-TODIM (P2TL-CPT-TODIM) method is defined to solve the MAGDM issues with P2TLSs; (3) the attribute weights are derived through the CRITIC method and CPT; (4) a case study for the performance evaluation of home-based elderly-care services purchased by the government is supplied to confirm the designed method; (5) some comparative analyses are supplied using existing methods to show the rationality of the P2TL-CPT-TODIM method; (6) the proposed P2TL-CPT-TODIM method not only enriches the decision-making method for all kinds of performance evaluation issues, but also demonstrates the potential role for the uncertain MAGDM algorithms in other fields.

The remainder of this paper is constructed as follows: In Section 2, a literature review regarding fuzzy sets is provided. In Section 3, we briefly review the basic concepts of P2TLNs. In Section 4, we briefly review the classical CPT-TODIM method. In Section 5, the CPT-TODIM method for MAGDM with P2TLNs is proposed. In Section 6, a case study for performance evaluations of home-based elderly-care services purchased by the government is given to demonstrate the proposed methods and compare them with existing decision methods with the aim of showing the availability of the proposed approach. Section 7 is the conclusion.

## 2. Literature Review

In numerous instances of MADM or MAGDM, information is described by crisp numbers. Thus, for decision-makers (DMs), most assessment information is imprecise or vague [24,25,26,27]. Hence, DMs cannot easily to deliver their preferences by taking advantage of an exact numerical value [28,29,30]. Atanassov [14] defined the concept of IFSs to simplistically describe the information of qualitative assessments. Hadjitodorov [31] designed the nearest prototype (NP) method within the IFS setting. Hung [32] defined a method to derive the partial correlation of IFSs by means of a multivariate correlation model. Xu and Yager [33] designed several geometric aggregation operators for IFSs. Zhou et al. [34] defined normalized weighted Bonferroni harmonic mean-based intuitionistic fuzzy operators for the sustainability of search and rescue robots. Cavallaro et al. [35] assessed the technologies of concentrated solar power (CSP) by using the IF-TOPSIS and trigonometric entropy weights. Garg [36] designed a novel strategy for solving IF-MADM issues that proposed using different entropies and unknown attribute weights. Liu and Li [37] defined some intuitionistic fuzzy Muirhead mean (IFMM) operators by extending MMs to IFNs. Lu and Wei [28] designed the TODIM method for performance appraisals of social-integration-based rural reconstruction under IVIFSs. Wu, Gao, and Wei [27] introduced the VIKOR method for financing risk assessment of rural tourism projects under IVIFSs. Wu et al. [38] proposed some interval-valued intuitionistic fuzzy Dombi Heronian mean operators for evaluating the ecological values of forest ecological tourism demonstration areas. Mohammadi and Makui [39] designed a new approach for supporting such decision situations based on IVIFSs and evidential reasoning. Wan et al. [40] proposed the intuitionistic fuzzy (IF) programming method to solve group decision-making (GDM) issues with IVFPRs, which derive the priority weights of alternatives under additive consistent IVFPR. Garg and Kumar [41] developed some novel similarity measures to depict the relative strength of the different IFSs after expressing the weakness of the existing measures. Kaur and Garg [42] built some new BMs and weighted BM-averaging operators between cubic IFNs to aggregate the different preferences of DMs. Wu et al. [43] designed algorithms for evaluating the competitiveness of tourist destinations using interval-valued intuitionistic fuzzy Hamy mean operators. Chen and Kuo [44] presented a novel MADM method along with a non-linear programming (NLP) methodology with the hyperbolic tangent function and IVIFVs. Chen et al. [45] designed a novel MADM methodology based on Shannon’s information entropy, non-linear programming (NLP) and IVIFVs, where attributes’ weights and evaluation attributes’ values with respect to alternatives were depicted by IVIFVs. Garg and Kumar [46] proposed the concept of linguistic-interval-valued Atanassov IFSs (LIVAIFSs) that could define relevant algorithms and related properties and propose a MADM example with a LIVAIF number. Arora and Garg [47] provided the related algorithms of linguistic intuitionistic fuzzy numbers, defined several weighted operators, discussed the properties of these operators, and verified them with an example. Wei [30] utilized arithmetic and geometric operations to propose some 2-tuple intuitionistic fuzzy linguistic aggregation operators.

Recently, Pythagorean fuzzy sets (PFSs) [16] have appeared as the valid means to describe MADM issues of uncertain information. In PFSs, two variable degrees are depicted—membership and non-membership degrees—whose sums of squares cannot exceed 1. Thus, PFSs are more general than IFSs. Zhang and Xu [48] designed the Pythagorean fuzzy number (PFN) concept then put forward PF-TOPSIS for MADM. Liang et al. [49] investigated the Pythagorean fuzzy Bonferroni mean (PFBM) and weighted PFBM (WPFBM) operator. Peng and Yang [50] designed a method that ranked the superiority and inferiority of PFNs in order to tackle MAGDM. Li and Lu [51] developed some new similarity and distance measures of PFSs. Garg [52] developed novel logarithm operational laws with a real number base lambda for the PFSs. Ren et al. [53] designed the PF-TODIM method, which takes into account the psychological behaviors of DMs under uncertain situations. Zeng et al. [54] brought forward a MAGDM framework based on PFSs that incorporated the self-confidence of decision-makers. Gul et al. [55] supplied with a theoretical contribution by suggesting a PF-VIKOR approach, and improved overall safety levels of underground mining by considering and advising on the potential hazards of practical risk-management applications. Yu et al. [56] investigated a new distance operator for IOWA in Pythagorean fuzzy MAGDM. Liang et al. [57] provided a compromised solution’s novel perspective that could tackle the psychological behaviors of DMs by integrating the TODIM and VIKOR methods. Zeb et al. [58] developed a credible extended Pythagorean fuzzy set (C-EPFS) and a possible extended Pythagorean fuzzy set (P-EPFS). Gou et al. [59] investigated the continuous PFNs’ properties. Liang et al. [60] studied the operators of Pythagorean fuzzy geometric BM (PFGBM) and weighted PFGBM (WPFGBM) in MCGDM. Chen [61] designed new PROMETHEE-based outranking algorithms for MCDA under PFSs. Thao and Smarandache [62] utilized a concept of probability to define the PFSs’ fuzzy entropy as an extension of the fuzzy entropy of IFSs. Chen [63] defined a consensus ranking method that used a mixed choice strategy for MCDA under a complex uncertainty that was based on PFSs. Teng et al. [64] developed some power MSM-fused operators for Pythagorean fuzzy linguistic information, including a fuzzy linguistic power MSM operator and Pythagorean fuzzy linguistic power-weighted MSM (PFLPWMSM) operator. Ma and Xu [65] modified the existing score function and accuracy function of PFNs and defined some operators including PFWA, PFWG, and others. Peng and Yang [66] defined integral Choquet operators for PFNs, such as the PFCIA and PFCIG operators, and developed two methods for MAGDM that considered the dependent and independent attributes of the foundations of the PFCIA operator and the MABAC model under a Pythagorean fuzzy environment. Garg [67] added fuzziness to the Pythagorean membership function to redefine the existing operation, thus proposing a Pythagorean theorem based on the Einstein norm operation and verifying the effectiveness of the new method through comparative analysis and examples. Li et al. [68] extended the Hamy mean (HM) operator and dual Hamy mean (DHM) operator with PFNs. Chen [69] built the novel correlation-based compromise method for addressing MCDA problems under complex uncertainty based on PFSs. Zhang [70] proposed a new PFN closeness index and introduced a PFN ranking method based on the closeness index. Garg [71] incorporated the confidence level of each PFN and studied some new average operators and geometric operators, namely, the confidence PFWA operator and the confidence PFWG operator, along with some of their desired properties. Wei et al. [72] extended the Pythagorean fuzzy set to the interval-valued Pythagorean fuzzy set, relaxed the input condition by using the Maclaurin symmetric mean (MSM) operator, put forward two kinds of operators to form a decision method, and finally applied it to a calculation example. Tang et al. [73] put forward some Pythagorean fuzzy Muirhead mean operators in MADM to evaluate the commercialization of emerging technology. Tang et al. [74] defined some MADM algorithms with Muirhead mean operators under IVPFSs. Geng et al. [75] proposed the Pythagorean fuzzy uncertain linguistic set and designed the extended TODIM method for MCGDM problems. Chen [76] defined the remoteness-index-based PF-VIKOR methods, which are significantly different from the existing VIKOR methods. Deng et al. [77] defined the generalized Heronian mean (GHM) operator, generalized weighted Heronian mean (GWHM), geometric Heronian mean (GHM) operator, and weighted geometric Heronian mean (WGHM) operator with 2-tuple linguistic Pythagorean fuzzy numbers (2TLPFNs). Garg and Harish [78] connected the Pythagorean fuzzy set with the linguistic fuzzy set. Wang et al. [79] extended the MSM operator, generalized MSM (GMSM), and dual MSM (DMSM) operator with IV2TLPFNs.

## 3. Preliminaries

### 3.1. Pythagorean 2-Tuple Linguistic Sets

Wei, Lu, Alsaadi, Hayat, and Alsaedi [17] defined P2TLSs based on the PFSs [18] and 2-tuple linguistic [19].

**Definition** **1** **([17]).***The Pythagorean 2-tuple linguistic set*P*in*X*is given as*

(1) $$P=\left\{\left(s_{\alpha (x)},\phi \right),\left(u_{p}\left(x\right),\nu_{p}\left(x\right)\right),x\in X\right\}$$

*where*$s_{\alpha (x)}\in S$*,*ϕ∈[−0.5,0.5),$u_{P}\left(x\right)\in [0,1]$*, and*$v_{P}\left(x\right)\in [0,1]$**,**$u_{p}\left(x\right)\;\mathrm{and}\;\nu_{p}\left(x\right)$*should meet the following condition:*$0\leq {\left(u_{P}\left(x\right)\right)}^{2}+{\left(v_{P}\left(x\right)\right)}^{2}\leq 1$*,*∀x∈X*. The functions*$u_{P}\left(x\right),\nu_{P}\left(x\right)$*refer to, accordingly, the membership degree and non-membership degree of the element*x*to the linguistic variable*$\left(s_{\alpha (x)},\phi \right)$*. For convenience, a Pythagorean 2-tuple linguistic number (P2TLN) denotes*$p=\langle \left(s_{p},\phi \right),\left(u_{p},v_{p}\right)\rangle$*.*

**Definition** **2** **([17]).***Assume that*$p=\langle \left(s_{p},\phi \right),\left(u_{p},v_{p}\right)\rangle$*is a P2TLN; the score function of P2TLN is*

(2) $$S(p)=\Delta \left(\Delta^{-1}\left(s_{\alpha \left(p\right)},\phi \right)\frac{1+{\left(u_{p}\right)}^{2}-{\left(\nu_{p}\right)}^{2}}{2}\right),\Delta^{-1}\left(S\left(p\right)\right)\in \left[1,t\right].$$

*where*Δ*is the function used to obtain the 2-tuple linguistic information equivalent to a numerical value (belonging to*[1,t]*), and*$\Delta^{-1}$*is the inverse operation of*Δ*.*

**Definition** **3** **([17]).***Assume that*$p=\langle \left(s_{p},\phi \right),\left(u_{p},v_{p}\right)\rangle$*is a P2TLN; the accuracy function of P2TLN is*

(3) $$H(p)=\Delta \left(\Delta^{-1}\left(s_{\alpha \left(p\right)},\phi \right)\frac{{\left(u_{p}\right)}^{2}+{\left(\nu_{p}\right)}^{2}}{2}\right),\Delta^{-1}\left(H\left(p\right)\right)\in \left[1,t\right].$$

**Definition** **4** **([34]).***Assume that*$p_{1}=\langle \left(s_{p_{1}},\phi_{1}\right),\left(u_{p_{1}},v_{p_{1}}\right)\rangle$*and*$p_{2}=\langle \left(s_{p_{2}},\phi_{2}\right),\left(u_{p_{2}},v_{p_{2}}\right)\rangle$*are two P2TLNs;*$S\left(p_{1}\right)=\Delta \left(\Delta^{-1}\left(s_{\alpha (p_{1})},\phi_{1}\right)\cdot \frac{1+{\left(u_{p_{1}}\right)}^{2}-{\left(\nu_{p_{1}}\right)}^{2}}{2}\right)$*and*$S\left(p_{2}\right)=\Delta \left(\Delta^{-1}\left(s_{\alpha (p_{2})},\phi_{2}\right)\cdot \frac{1+{\left(u_{p_{2}}\right)}^{2}-{\left(\nu_{p_{2}}\right)}^{2}}{2}\right)$*are the score values of*$p_{1}$*and*$p_{2}$*, respectively; and*$H\left(p_{1}\right)=\Delta \left(\Delta^{-1}\left(s_{\alpha (p_{1})},\phi_{1}\right)\cdot \frac{{\left(u_{p_{1}}\right)}^{2}+{\left(\nu_{p_{1}}\right)}^{2}}{2}\right)$*and*$H\left(p_{2}\right)=\Delta \left(\Delta^{-1}\left(s_{\alpha (p_{2})},\phi_{2}\right)\cdot \frac{{\left(u_{p_{2}}\right)}^{2}+{\left(\nu_{p_{2}}\right)}^{2}}{2}\right)$*are the accuracy values of*$p_{1}$*and*$p_{2}$*. This being so,*

$\left(1\right)if\;S\left(p_{1}\right)<S\left(p_{2}\right),p_{1}<p_{2};$
$\left(2\right)if\;S\left(p_{1}\right)>S\left(p_{2}\right),p_{1}>p_{2};$
$(3)\;if\;S\left(p_{1}\right)=S\left(p_{2}\right),H\left(p_{1}\right)<H\left(p_{2}\right),\;then\;p_{1}<p_{2};$
$(3)\;if\;S\left(p_{1}\right)=S\left(p_{2}\right),H\left(p_{1}\right)>H\left(p_{2}\right),\;then\;p_{1}>p_{2};$
$(3)\;if\;S\left(p_{1}\right)=S\left(p_{2}\right),H\left(p_{1}\right)=H\left(p_{2}\right),\;then\;p_{1}=p_{2};$

Wei, Lu, Alsaadi, Hayat, and Alsaedi [17] defined some operational laws of P2TLNs.

**Definition** **5.** *Assume that*$p_{1}=\langle \left(s_{p_{1}},\phi_{1}\right),\left(u_{p_{1}},v_{p_{1}}\right)\rangle$*and*$p_{2}=\langle \left(s_{p_{2}},\phi_{2}\right),\left(u_{p_{2}},v_{p_{2}}\right)\rangle$*are two P2TLNs; the normalized Hamming distance between*$p_{1}$*and*$p_{2}$*is defined as*

(4) $$d\left(p_{1},p_{2}\right)=\frac{1}{2L}\left[\left|\begin{array}{l} \left(1+{\left(u_{p_{1}}\right)}^{2}-{\left(v_{p_{1}}\right)}^{2}\right)\cdot \Delta^{-1}\left(s_{p_{1}},\phi_{1}\right)-\\ \left(1+{\left(u_{p_{2}}\right)}^{2}-{\left(v_{p_{2}}\right)}^{2}\right)\cdot \Delta^{-1}\left(s_{p_{2}},\phi_{2}\right) \end{array}\right|\right]$$

*where L is a value of a number on behalf of the length of the language scale.*

**Definition** **6** **([17]).***Assume that*$p_{1}=\langle \left(s_{p_{1}},\phi_{1}\right),\left(u_{p_{1}},v_{p_{1}}\right)\rangle$*and*$p_{2}=\langle \left(s_{p_{2}},\phi_{2}\right),\left(u_{p_{2}},v_{p_{2}}\right)\rangle$*are two P2TLNs; then,*

$$\begin{array}{l} p_{1}⊕p_{2}=\langle \Delta \left(\Delta^{-1}\left(s_{p_{1}},\phi_{1}\right)+\Delta^{-1}\left(s_{p_{2}},\phi_{2}\right)\right),\left(\sqrt{{\left(u_{p_{1}}\right)}^{2}+{\left(u_{p_{2}}\right)}^{2}-{\left(u_{p_{1}}\right)}^{2}{\left(u_{p_{2}}\right)}^{2}},\nu_{p_{1}}\nu_{p_{2}}\right)\rangle ;\\ p_{1}⊗p_{2}=\langle \Delta \left(\Delta^{-1}\left(s_{p_{1}},\phi_{1}\right)\cdot \Delta^{-1}\left(s_{p_{2}},\phi_{2}\right)\right),\left(u_{p_{1}}u_{p_{2}},\sqrt{{\left(\nu_{p_{1}}\right)}^{2}+{\left(\nu_{p_{2}}\right)}^{2}-{\left(\nu_{p_{1}}\right)}^{2}{\left(\nu_{p_{2}}\right)}^{2}}\right)\rangle ;\\ \lambda p_{1}=\langle \Delta \left(\lambda \Delta^{-1}\left(s_{p_{1}},\phi_{1}\right)\right),\left(\sqrt{1-{\left(1-{\left(u_{p_{1}}\right)}^{2}\right)}^{\lambda }},{\left(\nu_{p_{1}}\right)}^{\lambda }\right)\rangle ;\\ {\left(p_{1}\right)}^{\lambda }=\langle \Delta \left({\left(\Delta^{-1}\left(s_{p_{1}},\phi_{1}\right)\right)}^{\lambda }\right),\left({\left(u_{p_{1}}\right)}^{\lambda },\sqrt{1-{\left(1-{\left(\nu_{p_{1}}\right)}^{2}\right)}^{\lambda }}\right)\rangle . \end{array}$$

### 3.2. P2TLWA and P2TLWG Operators

In this part, we introduce some aggregation operators that use P2TLNs.

**Definition** **7** **([17]).***Assume that*$p=\langle \left(s_{p_{j}},\phi_{j}\right),\left(u_{p_{j}},v_{p_{j}}\right)\rangle \left(j=1,2,\ldots ,n\right)$*is a set of P2TLNs; the Pythagorean 2-tuple linguistic weighted averaging (P2TLWA) operator can be defined as follows:*

(5) $$\begin{array}{l} {P2\mathrm{TLWA}}_{\omega }\left(p_{1},p_{2},\ldots ,p_{n}\right)=\overset{n}{\underset{j=1}{⊕}}\left(\omega_{j}p_{j}\right)\\ =\langle \Delta \left(\sum_{j=1}^{n}\omega_{j}\Delta^{-1}\left(s_{p_{j}},\phi_{j}\right)\right),\left(\sqrt{1-\prod_{j=1}^{n}{\left(1-{\left(u_{p_{j}}\right)}^{2}\right)}^{\omega_{j}}},\prod_{j=1}^{n}{\left(\nu_{p_{j}}\right)}^{\omega_{j}}\right)\rangle \end{array}$$

*where*$\omega ={\left(\omega_{1},\omega_{2},\ldots ,\omega_{n}\right)}^{T}$*is the weight vector of*$p_{j}\left(j=1,2,\ldots ,n\right)$*with*$\omega_{j}>0,\sum_{j=1}^{n}\omega_{j}=1.$

**Definition** **8** **([17]).***Assume that*$p=\langle \left(s_{p_{j}},\phi_{j}\right),\left(u_{p_{j}},v_{p_{j}}\right)\rangle \left(j=1,2,\ldots ,n\right)$*is a set of P2TLNs; the Pythagorean 2-tuple linguistic weighted geometric (P2TLWG) operator can be defined as follows:*

(6) $$\begin{array}{l} {P2\mathrm{TLWG}}_{\omega }\left(p_{1},p_{2},\ldots ,p_{n}\right)=\overset{n}{\underset{j=1}{⊗}}\left(\omega_{j}p_{j}\right)\\ =\langle \Delta \left(\prod_{j=1}^{n}\Delta^{-1}{\left(s_{p_{j}},\phi_{j}\right)}^{\omega_{j}}\right),\left(,\prod_{j=1}^{n}{\left(u_{p_{j}}\right)}^{\omega_{j}}\sqrt{1-\prod_{j=1}^{n}{\left(1-{\left(\nu_{p_{j}}\right)}^{2}\right)}^{\omega_{j}}}\right)\rangle \end{array}$$

*where*$\omega ={\left(\omega_{1},\omega_{2},\ldots ,\omega_{n}\right)}^{T}$*is the weight vector of*$p_{j}\left(j=1,2,\ldots ,n\right)$*with*$\omega_{j}>0,\sum_{j=1}^{n}\omega_{j}=1.$

## 4. The CPT-TODIM Method

The main contribution of this article is to replace the original weight of the extended TODIM proposed by Tian, Xu, and Gu [13] with the CPT weight function and modify the perceived value of dominance. The general process of implementing the CPT-TODIM method is depicted in the following section.

**Step 1**: Set up a panel of DMs, choose the appropriate criteria, screen the prospective alternatives out for the MADM problem, and finally form the decision matrix $P={\left(p_{ij}\right)}_{m\times n}$, where $p_{ij}$ is the possible value of the alternative evaluation $A_{i}\left(i=1,2,\ldots ,m\right)$ in regards to attribute $\xi_{j}\left(j=1,2,\ldots ,n\right)$ according to DMs, m means the number of alternatives, and n means the number of criteria. 

**Step 2:** Normalize the decision matrix $P={\left(p_{ij}\right)}_{m\times n}$ into $C={\left(c_{ij}\right)}_{m\times n}.$

(7) $$c_{ij}=\{\begin{array}{c} p_{ij}\\ -p_{ij} \end{array}\;\;\;\begin{array}{c} if\;\xi_{j}\;\mathrm{is}\;\mathrm{benefit}\;\mathrm{attribute},\;\\ if\;\xi_{j}\;\mathrm{is}\;\cos t\;\mathrm{attribute}.\; \end{array}$$

Here, the normalization method should be determined according to different fuzzy environments.

**Step 3:** Work out the transformed probability of the alternative $A_{i}$ to $A_{k}$(i≠k).

When $x_{ij}-x_{kj}\geq 0$, the transformed probability weight is acquired using Equation (8):

(8) $$\pi_{ikj}^{+}\left(\omega_{j}\right)={\omega_{j}^{\kappa }/\left(\omega_{j}^{\kappa }+{\left(1-\omega_{j}\right)}^{\kappa }\right)}^{\frac{1}{\kappa }}$$

When $x_{ij}-x_{kj}<0$, the transformed probability weight is acquired using Equation (9):

(9) $$\pi_{ikj}^{-}\left(\omega_{j}\right)={\omega_{j}^{\lambda }/\left(\omega_{j}^{\lambda }+{\left(1-\omega_{j}\right)}^{\lambda }\right)}^{\frac{1}{\lambda }}$$

where κ and λ are parameters equaling 0.61 and 0.69, respectively.

**Step 4:** Determine the relative weight $\pi_{ikj}{}^{*}$ for the alternative $A_{i}$ to the alternative $A_{k}$ using Equation (10):

(10) $$\pi_{ikj}{}^{*}=\pi_{ikj}\left(\omega_{j}\right)/\pi_{ikr}\left(\omega_{r}\right)\;\;\;\;\;\;\;\;\;\;\;\;\;\;\;\;\;\;\;\;\;\;\;\;\;\;\;\;\;\;\;\;\;\;\;\;\;\;\;\;\;\;r,j\in M,\forall \left(i,k\right)\;$$

where $\pi_{ikj}\left(\omega_{j}\right)$ and $\pi_{ikr}\left(\omega_{r}\right)\;\;$ are acquired from Equation (8) or (9) for the alternative $A_{i}$ to $A_{k}$ depending on the value of $x_{ij}-x_{kj}$; $\pi_{ikj}\left(\omega_{j}\right)$ represents the transformed weight of the jth attribute for the alternative $A_{i}$; and $\pi_{ikr}\left(\omega_{r}\right)\;\;$ refers to the transformed weight of reference attribute for the alternative $A_{i}$ to $A_{k}$, satisfying $\pi_{ikr}\left(\omega_{r}\right)\;\;=\max\left(\pi_{ikj}\left(\omega_{j}\right)|j\in M\right)$.

**Step 5:** Calculate the relative prospect dominance of the alternative $A_{i}$ to $A_{k}$ under attribute j using Equation (11):

(11) $$\varphi_{j}{}^{*}\left(A_{i},A_{k}\right)=\left\{ \begin{array}{ll} \pi_{ikj}{}^{*}{\left(x_{ij}-x_{kj}\right)}^{\alpha }/\sum_{j^{*}=1}^{m}\pi_{ikj}{}^{*}, & if\;x_{ij}>\;x_{kj}\\ 0, & if\;x_{ij}=\;x_{kj}\\ -\vartheta \left(\sum_{j^{*}=1}^{m}\pi_{ikj}{}^{*}\right){\left(x_{ij}-x_{kj}\right)}^{\beta }/\pi_{ikj}{}^{*} & if\;x_{ij}<\;x_{kj} \end{array}\right.$$

where α, β, and ϑ are parameters.

**Step 6:** Aggregate the relative prospect dominance of the alternative $A_{i}$ to $A_{k}$ under all the attributes using Equation (12):

(12) $$\phi \left(A_{i},A_{k}\right)=\sum_{j^{*}=1}^{m}\varphi_{j}{}^{*}\left(A_{i},A_{k}\right),\;\;\;\;\;\;\;\;\;\;\forall \left(i,k\right)$$

**Step 7:** Obtain the overall prospect dominance of the alternative $A_{i}$ based on Equation (12).

**Step 8:** Rank the overall prospect dominance $\phi \left(A_{i}\right)$,i∈N, based on which optimal alternative is found. The bigger the overall prospect value $\phi \left(A_{i}\right)$ is, the better project $A_{i}$ will be.

## 5. The CPT-TODIM Method with P2TLNs

In this section, we combine this extended TODIM method with P2TLNs and detail all the steps to try and address the MAGDM problems. Suppose there are m alternatives $\left\{A_{1},A_{2},\ldots A_{m}\right\}$, n attributes $\left\{\xi_{1},\xi_{2},\ldots \xi_{n}\right\}$, and l experts $\left\{E_{1},E_{2},\ldots E_{l}\right\}$. Let $\left\{\lambda_{1},\lambda_{2},\ldots \lambda_{l}\right\}$ and $\left\{\omega_{1},\omega_{2},\ldots \omega_{n}\right\}$ be the expert’s weighting vector and attribute’s weighting vector, which satisfy $\lambda_{k}\in \left[0,1\right],\omega_{j}\in \left[0,1\right]$ and $\sum_{k=1}^{l}\lambda_{k}=1,\sum_{j=1}^{n}\omega_{j}=1$. The calculation steps are as follows.

**Step 1:** Set up a panel of DMs, choose the appropriate criteria, screen the prospective alternatives out for the MADM problem, and form the P2TLN decision matrix $P^{\left(k\right)}={\left(p_{ij}^{k}\right)}_{m\times n}$ for each decision-maker to compute the group P2TLN decision matrix $P={\left(p_{ij}\right)}_{m\times n}$.

(13) $$P^{\left(k\right)}={\left[p_{ij}^{k}\right]}_{m\times n}=\left[\begin{array}{cccc} p_{11}^{k} & p_{12}^{k} & \ldots & p_{1n}^{k}\\ p_{21}^{k} & p_{22}^{k} & \ldots & p_{2n}^{k}\\ \vdots & \vdots & \vdots & \vdots \\ p_{m1}^{k} & p_{m2}^{k} & \ldots & p_{mn}^{k} \end{array}\right]$$

(14) $$P={\left[pij\right]}_{m\times n}=\left[\begin{array}{cccc} p_{11} & p_{12} & \ldots & p_{1n}\\ p_{21} & p_{22} & \ldots & p_{2n}\\ \vdots & \vdots & \vdots & \vdots \\ p_{m1} & p_{m2} & \ldots & p_{mn} \end{array}\right]$$

(15) $$\begin{array}{l} p_{ij}=\overset{l}{\underset{k=1}{⊕}}p_{ij}^{k}=P2\mathrm{TLWA}(p_{ij}^{1},p_{ij}^{2},\ldots ,p_{ij}^{l})\\ =\langle \Delta \left(\sum_{k=1}^{l}\lambda_{k}\Delta^{-1}\left(s_{p_{ij}},\phi_{ij}\right)\right),\left(\sqrt{1-\prod_{k=1}^{l}{\left(1-{\left(u_{ij}^{k}\right)}^{2}\right)}^{\lambda_{k}}},\prod_{k=1}^{l}{\left(\nu_{ij}^{k}\right)}^{\lambda_{k}}\right)\rangle \end{array}$$

where $p_{ij}^{k}$ is the possible value of the alternative evaluation $\eta_{i}\left(i=1,2,\ldots ,m\right)$ in regard to attribute $\xi_{j}\left(j=1,2,\ldots ,n\right)$ according to DMs $p_{k}\left(k=1,2,\ldots ,l\right)$; m means the number of alternatives; n means the number of criteria; and k denotes the number of decision-makers. 

**Step 2:** Normalize the decision matrix $P={\left(p_{ij}\right)}_{m\times n}$ into $C={\left(c_{ij}\right)}_{m\times n}.$

(16) $$c_{ij}=\{\begin{array}{c} \langle \left(s_{p_{{}_{ij}}},\phi_{ij}\right),\left(u_{ij},v_{ij}\right)\rangle \\ \langle \Delta \left(L-\Delta^{-1}\left(s_{p_{ij}},\phi_{ij}\right)\right),\left(v_{ij},u_{ij}\right)\rangle \end{array}\;\;\;\begin{array}{c} if\;\xi_{j}\;\mathrm{is}\;\mathrm{benefit}\;\mathrm{attribute},\;\\ if\;\xi_{j}\;\mathrm{is}\;\mathrm{cost}\;\mathrm{attribute}.\; \end{array}$$

**Step 3:** Compute the weights of attributes by using the CRITIC method.

CRiteria Importance Through Intercriteria Correlation (CRITIC) is introduced to determine the weights of attributes. Diakoulaki et al. [80] initially presented this method to take the correlations between attributes into account. In this method, the attributes are not in contradiction with each other, and the attribute weights are determined using the decision matrix. Below, we describe the computational steps of this method.

**Step 1:** Based on the normalized matrix $C={\left(c_{ij}\right)}_{m\times n}$, the correlation coefficient among attributes is determined using Equation (17)

(17) $$c_{jt}=\frac{\sum_{i=1}^{m}\left(S\left(c_{ij}\right)-S\left(c_{j}\right)\right)\left(S\left(c_{it}\right)-S\left(c_{t}\right)\right)}{\sqrt{\sum_{i=1}^{m}{\left(S\left(c_{ij}\right)-S\left(c_{j}\right)\right)}^{2}}\sqrt{\sum_{i=1}^{m}{\left(S\left(c_{it}\right)-S\left(c_{t}\right)\right)}^{2}}}\;\;\;\;\;\;\;j,t\in \left(1,n\right)$$

where $S\left(c_{j}\right)=\frac{1}{m}\sum_{i=1}^{m}S\left(c_{ij}\right)$ and $S\left(c_{t}\right)=\frac{1}{m}\sum_{i=1}^{m}S\left(c_{it}\right)$

**Step 2:** Compute the standard deviation of the attribute.

(18) $$std_{j}=\sqrt{\frac{1}{m-1}\sum_{i=1}^{m}{\left(S\left(c_{ij}\right)-S\left(c_{j}\right)\right)}^{2}}\;\;\;\;\;\;j\in \left(1,n\right)$$

**Step 3:** The weights of attributes are determined by Equation (19).

(19) $$\omega_{j}=\frac{std_{j}\sum_{t=1}^{n}\left(1-c_{jk}\right)}{\sum_{j=1}^{n}\left(std_{j}\sum_{t=1}^{n}\left(1-c_{jk}\right)\right)}\;\;\;\;\;\;\;j,t\in \left(1,n\right)$$

where $\omega_{j}\in \left[0,1\right]$ and $\sum_{j=1}^{n}\omega_{j}=1$.

**Step 4:** Work out the transformed probability of the alternative $A_{i}$ to $A_{k}$(i≠k).

When $x_{ij}-x_{kj}\geq 0$, the transformed probability weight is acquired using Equation (20):

(20) $$\pi_{ikj}^{+}\left(\omega_{j}\right)={\omega_{j}^{\kappa }/\left(\omega_{j}^{\kappa }+{\left(1-\omega_{j}\right)}^{\kappa }\right)}^{\frac{1}{\kappa }}$$

When $x_{ij}-x_{kj}<0$, the transformed probability weight is acquired using (21):

(21) $$\pi_{ikj}^{-}\left(\omega_{j}\right)={\omega_{j}^{\lambda }/\left(\omega_{j}^{\lambda }+{\left(1-\omega_{j}\right)}^{\lambda }\right)}^{\frac{1}{\lambda }}$$

Where κ and λ are parameters.

**Step 5:** Determine the relative weight $\pi_{ikj}{}^{*}$ for the alternative $A_{i}$ to the alternative $A_{k}$ using (22):

(22) $$\pi_{ikj}{}^{*}=\pi_{ikj}\left(\omega_{j}\right)/\pi_{ikr}\left(\omega_{r}\right)\;\;\;\;\;\;\;\;\;\;\;\;\;\;\;\;\;\;\;\;\;\;\;\;\;\;\;\;\;\;\;\;\;\;\;\;\;\;\;\;\;\;r,j\in M,\forall \left(i,k\right)\;$$

where $\pi_{ikj}\left(\omega_{j}\right)$ and $\pi_{ikr}\left(\omega_{r}\right)\;\;$ are acquired from Equations (20) and (21), respectively, for the alternative $A_{i}$ to $A_{k}$ depending on the value of $x_{ij}-x_{kj}$; $\pi_{ikj}\left(\omega_{j}\right)$ represents the transformed weight of the jth attribute for the alternative $A_{i}$; and $\pi_{ikr}\left(\omega_{r}\right)\;\;$ refers to the transformed weight of the reference attribute for the alternative $A_{i}$ to $A_{k}$, satisfying $\pi_{ikr}\left(\omega_{r}\right)\;\;=\max\left(\pi_{ikj}\left(\omega_{j}\right)|j\in M\right)$.

**Step 6:** Calculate the relative prospect dominance of the alternative $A_{i}$ to $A_{k}$ under attribute j using Equation (23):

(23) $$\varphi_{j}{}^{*}\left(A_{i},A_{k}\right)=\left\{ \begin{array}{cc} \pi_{ikj}{}^{*}{\left(x_{ij}-x_{kj}\right)}^{\alpha }/\sum_{j^{*}=1}^{m}\pi_{ikj}{}^{*}, & if\;x_{ij}>\;x_{kj}\\ 0, & if\;x_{ij}=\;x_{kj}\\ -\vartheta \left(\sum_{j^{*}=1}^{m}\pi_{ikj}{}^{*}\right){\left(x_{ij}-x_{kj}\right)}^{\beta }/\pi_{ikj}{}^{*} & if\;x_{ij}<\;x_{kj} \end{array}\right.$$

where α, β, and ϑ are parameters.

**Step 7:** Aggregate the relative prospect dominance of the alternative $A_{i}$ to $A_{k}$ under all the attributes using Equation (24):

(24) $$\phi \left(A_{i},A_{k}\right)=\sum_{j^{*}=1}^{m}\varphi_{j}{}^{*}\left(A_{i},A_{k}\right),\;\;\;\;\;\;\;\;\;\;\forall \left(i,k\right)$$

**Step 8:** Obtain the overall prospect dominance of the alternative $A_{i}$ using Equation (24).

**Step 9:** Rank the overall prospect dominance $\phi \left(A_{i}\right)$, i∈N, based on which the optimal alternative is then found. The bigger the overall prospect value $\phi \left(A_{i}\right)$ is, the better project $A_{i}$ will be.

## 6. Numerical Example and Comparative Analysis

### 6.1. Numerical Example

At present, the institutional transference represented by the governmental purchasing of private organizations including home endowments is successful in most parts of the country, and evaluation of this community home endowment service is discussed in this section. Performance evaluations of home-based elderly-care services that have been purchased by the government represent a classical MADM issue [81,82,83,84]. Thus, we shall use P2TLNs to depict the assessment information and use the P2TL-CPT-TODIM model for a performance evaluation. Suppose that there are five home-based elderly-care care projects $A_{i}\left(i=1,2,3,4,5\right)$ to be selected, and five evaluation attributes $\xi_{j}\left(j=1,2,3,4\right)$ that are utilized to assess these home-based care projects: ①$\xi_{1}$ is the timely availability of government funds; ②$\xi_{2}$ is the government funds to determine the strength of the project; ③$\xi_{3}$ is the soundness of the project management system; ④$\xi_{4}$ is implementation of the project management system; ⑤$\xi_{5}$ is elderly satisfaction. The five possible projects $A_{i}\left(i=1,2,3,4,5\right)$ are evaluated with P2TLNs in regard to the four attributes by five experts $E^{k}$(expert’s weight λ=(0.23,0.26,0.18,0.16,0.17), attribute’s weight unknown).

Follow these steps to evaluate the five home-care programs.

**Step 1:** Construct the evaluation matrix $P^{\left(k\right)}={\left(p_{ij}^{k}\right)}_{5\times 5}\left(i=1,2,\ldots ,5,j=1,2,\ldots ,5\right)$ for each DM listed in Table 1, Table 2, Table 3, Table 4 and Table 5. Based on Table 1, Table 2, Table 3, Table 4 and Table 5 and Equation (14), the group decision matrix is computed and presented in Table 6.

**Step 2:** Because all attributes are beneficial, there is no need for normalization.

**Step 3:** Compute the weights of attributes using the CRITIC method. The correlation coefficient matrix among attributes is computed according to Equation (17), as in Table 7. The standard deviation of each attribute is estimated according to Equation (18), as in Table 8. The weights of attributes are determined by Equation (19), as shown in Table 9.

Step 4: Calculate the transformed probability of the alternative $A_{i}$ to $A_{k}$ according to Equations (19) and (20), and derive the relative weight $\pi_{ikj}{}^{*}$ for the alternative $A_{i}$ to the alternative $A_{k}$. The results are depicted as in the Table 10, Table 11, Table 12, Table 13 and Table 14, where the values of the two parameters κ and λ are 0.61 and 0.69, respectively:

**Step 5:** Calculate the relative prospect dominance of the alternative $A_{i}$ to $A_{k}$ using Equation (22), as shown in Table 15, Table 16, Table 17, Table 18 and Table 19, where α=0.88, β=0.88, and ϑ=2.25.

**Step 6:** Aggregate the relative prospect dominance of the alternative $A_{i}$ to $A_{k}$ for all the attributes. The sum result is depicted in Table 20.

So, $A_{5}$ is the best alternative. 

### 6.2. Comparative Analysis

#### 6.2.1. Compared to P2TLWA/ P2TLWG operator

We make a comparison between our proposed method and other existing operators, including the P2TLWA/P2TLWG operator [17]. The results are shown in Table 21 and Table 22.

#### 6.2.2. Compared to P2TL-TODIM method

The TODIM method for the Pythagorean 2-tuple linguistic (P2TL-TODIM) [20] is processed for the sake of comparing it with the extended one. The calculating result of P2TL-TODIM is listed in Table 23. In order to compare these two methods more conveniently, the decision-making information in Table 1 and Table 2 is adopted here as well.

From the above comparison results, we can draw two conclusions. First, the comparison with P2TLWA/P2TLWG (the two original operators) shows that the ordering results from the two methods are the same. A5 is considered to be the best project to provide home-based elderly-care services, which verifies the stability and accuracy of this method. Second, compared with the traditional P2TL-TODIM, although the ranking results are consistent, the biggest difference of our new method lies in the application of different weighting functions and value functions, and the overall advantages obtained by the two methods are also different, making it more reasonable and scientific to apply to practical MAGDM problems.

## 7. Conclusions

The traditional MADM or MAGDM methods using P2TLNs have focused on decision-making with an assumption of perfect rationality. However, these existing methods and algorithms have seldom paid attention to the irrational characteristics of DMs, which are always meaningful to the assessment information. Although the TODIM method is a useful tool for simulating the irrational parts of DMs, it does not reflect the overall psychological states of DMs explained in CPT. Hence, in this study, we extended the classical TODIM method on the basis of a prospective value in CPT to solve the MAGDM issues with P2TLNs for the sake of comprehensively handling the irrational decision-making of DMs. Finally, the proposed method, named the P2TL-CPT-TODIM method, was proven superior to the classical method using a case study evaluation the performance of a home-based elderly-care service purchased by the government. 

The main contribution of this paper lies in the design of a performance evaluation method that uses the CPT-TODIM method to qualitatively evaluate the performance of government when purchasing a home-based service using P2TLSs. The method has some significant advantages: (1) the TODIM is modified based on the cumulative prospect theory (CPT) using P2TLSs; (2) the Pythagorean 2-tuple linguistic CPT-TODIM (P2TL-CPT-TODIM) method is designed to tackle the MAGDM issues with P2TLSs; (3) the attribute weights are obtained based on the CRITIC method and CPT; (4) a case study of a performance evaluation of a home-based elderly-care service purchased by the government is used to prove the developed method; (5) some comparative studies are given with existing methods to verify the rationality of the P2TL-CPT-TODIM method.

Although the extended P2TL-CPT-TODIM has been well applied to a performance evaluation of a home-based elderly-care service purchased by the government, we ignored the application of DM’ psychology to other fields. Furthermore, we believe that this study may provide inspiration for follow-up research of other MADM or MAGDM methods under the framework of bounded rationality. In the future, we will focus on extending the MADM and MAGDM methods under a fuzzy decision-making setting with the bounded rationality of DMs. At the same time, with the progress of society and the arrival of internet plus, government purchases of home-based services will continue to be given new content and form. How to increase the evaluation index, and how to accurately assess the indicators and compare with other methods such as DEA [4] and the P2TLN-Taxonomy [85] are interesting topics for our future studies. At the same time, the proposed models and algorithms could also been extended to other evaluation issues in social domains [86,87,88,89,90,91,92] as well as other applicable issues [91,92,93,94,95].

## Figures and Tables

**Table 1:** P2TLN decision matrix by DM_1_.

	$\xi_{1}$	$\xi_{2}$	$\xi_{3}$	$\xi_{4}$	$\xi_{5}$
$A_{1}$	<(s_5_,0), (0.8,0.5)>	<(s_1_,0), (0.1,0.9)>	<(s_3_,0), (0.5,0.1)>	<(s_2_,0), (0.3,0.7)>	<(s_1_,0), (0.4,0.4)>
$A_{2}$	<(s_0_,0), (0.3,0.1)>	<(s_2_,0), (0.5,0.6)>	<(s_1_,0), (0.9,0.8)>	<(s_6_,0), (0.5,0.3)>	<(s_4_,0), (0.1,0.5)>
$A_{3}$	<(s_1_,0), (0.6,0.7)>	<(s_6_,0), (0.8,0.3)>	<(s_0_,0), (0.1,0.9)>	<(s_1_,0), (0.9,0.2)>	<(s_0_,0), (0.8,0.9)>
$A_{4}$	<(s_2_,0), (0.6,0.5)>	<(s_2_,0), (0.6,0.4)>	<(s_5_,0), (0.7,0.4)>	<(s_5_,0), (0.1,0.6)>	<(s_2_,0), (0.6,0.5)>
$A_{5}$	<(s_2_,0), (0.8,0.8)>	<(s_6_,0), (0.6,0.9)>	<(s_2_,0), (0.4,0.5)>	<(s_4_,0), (0.6,0.7)>	<(s_4_,0), (0.9,0.6)>

**Table 2:** P2TLN decision matrix by DM_2_.

	$\xi_{1}$	$\xi_{2}$	$\xi_{3}$	$\xi_{4}$	$\xi_{5}$
$A_{1}$	<(s_2_,0), (0.5,0.8)>	<(s_2_,0), (0.1,0.5)>	<(s_2_,0), (0.7,0.1)>	<(s_6_,0), (0.1,0.9)>	<(s_3_,0), (0.1,0.7)>
$A_{2}$	<(s_5_,0), (0.1,0.8)>	<(s_2_,0), (0.4,0.9)>	<(s_5_,0), (0.6,0.9)>	<(s_0_,0), (0.4,0.8)>	<(s_5_,0), (0.3,0.5)>
$A_{3}$	<(s_0_,0), (0.5,0.7)>	<(s_4_,0), (0.1,0.4)>	<(s_0_,0), (0.2,0.4)>	<(s_2_,0), (0.4,0.7)>	<(s_1_,0), (0.7,0.1)>
$A_{4}$	<(s_6_,0), (0.5,0.3)>	<(s_1_,0), (0.6,0.2)>	<(s_6_,0), (0.1,0.3)>	<(s_6_,0), (0.4,0.4)>	<(s_3_,0), (0.9,0.4)>
$A_{5}$	<(s_6_,0), (0.5,0.5)>	<(s_3_,0), (0.9,0.2)>	<(s_1_,0), (0.5,0.7)>	<(s_5_,0), (0.6,0.1)>	<(s_5_,0), (0.7,0.7)>

**Table 3:** P2TLN decision matrix by DM_3_.

	$\xi_{1}$	$\xi_{2}$	$\xi_{3}$	$\xi_{4}$	$\xi_{5}$
$A_{1}$	<(s_4_,0), (0.9,0.5)>	<(s_6_,0), (0.9,0.3)>	<(s_4_,0), (0.1,0.8)>	<(s_5_,0), (0.7,0.1)>	<(s_4_,0), (0.9,0.7)>
$A_{2}$	<(s_1_,0), (0.7,0.9)>	<(s_2_,0), (0.5,0.3)>	<(s_3_,0), (0.2,0.7)>	<(s_1_,0), (0.7,0.5)>	<(s_6_,0), (0.4,0.8)>
$A_{3}$	<(s_3_,0), (0.5,0.3)>	<(s_2_,0), (0.7,0.1)>	<(s_0_,0), (0.3,0.3)>	<(s_0_,0), (0.5,0.1)>	<(s_2_,0), (0.7,0.1)>
$A_{4}$	<(s_5_,0), (0.1,0.4)>	<(s_1_,0), (0.8,0.9)>	<(s_2_,0), (0.2,0.5)>	<(s_2_,0), (0.1,0.6)>	<(s_5_,0), (0.7,0.1)>
$A_{5}$	<(s_3_,0), (0.9,0.2)>	<(s_0_,0), (0.9,0.6)>	<(s_0_,0), (0.2,0.4)>	<(s_1_,0), (0.8,0.7)>	<(s_4_,0), (0.9,0.2)>

**Table 4:** P2TLN decision matrix by DM_4_.

	$\xi_{1}$	$\xi_{2}$	$\xi_{3}$	$\xi_{4}$	$\xi_{5}$
$A_{1}$	<(s_1_,0), (0.2,0.1)>	<(s_6_,0), (0.7,0.3)>	<(s_0_,0), (0.3,0.4)>	<(s_1_,0), (0.9,0.5)>	<(s_1_,0), (0.1,0.1)>
$A_{2}$	<(s_0_,0), (0.1,0.1)>	<(s_3_,0), (0.7,0.3)>	<(s_3_,0), (0.1,0.8)>	<(s_0_,0), (0.9,0.1)>	<(s_6_,0), (0.7,0.9)>
$A_{3}$	<(s_6_,0), (0.2,0.9)>	<(s_3_,0), (0.1,0.4)>	<(s_1_,0), (0.6,0.7)>	<(s_3_,0), (0.1,0.3)>	<(s_3_,0), (0.3,0.3)>
$A_{4}$	<(s_4_,0), (0.3,0.2)>	<(s_3_,0), (0.2,0.6)>	<(s_6_,0), (0.3,0.5)>	<(s_0_,0), (0.9,0.9)>	<(s_6_,0), (0.2,0.1)>
$A_{5}$	<(s_5_,0), (0.7,0.2)>	<(s_6_,0), (0.2,0.1)>	<(s_4_,0), (0.5,0.3)>	<(s_6_,0), (0.2,0.3)>	<(s_2_,0), (0.6,0.2)>

**Table 5:** P2TLN decision matrix by DM_5._

	$\xi_{1}$	$\xi_{2}$	$\xi_{3}$	$\xi_{4}$	$\xi_{5}$
$A_{1}$	<(s_0_,0), (0.4,0.9)>	<(s_4_,0), (0.4,0.5)>	<(s_2_,0), (0.1,0.6)>	<(s_4_,0), (0.4,0.7)>	<(s_2_,0), (0.8,0.6)>
$A_{2}$	<(s_3_,0), (0.1,0.8)>	<(s_2_,0), (0.8,0.2)>	<(s_5_,0), (0.1,0.8)>	<(s_6_,0), (0.5,0.2)>	<(s_5_,0), (0.7,0.1)>
$A_{3}$	<(s_2_,0), (0.9,0.2)>	<(s_5_,0), (0.1,0.1)>	<(s_0_,0), (0.1,0.4)>	<(s_0_,0), (0.5,0.5)>	<(s_5_,0), (0.1,0.6)>
$A_{4}$	<(s_1_,0), (0.1,0.9)>	<(s_0_,0), (0.3,0.5)>	<(s_4_,0), (0.9,0.2)>	<(s_1_,0), (0.4,0.1)>	<(s_5_,0), (0.1,0.5)>
$A_{5}$	<(s_5_,0), (0.3,0.1)>	<(s_3_,0), (0.5,0.5)>	<(s_2_,0), (0.3,0.5)>	<(s_2_,0), (0.9,0.9)>	<(s_0_,0), (0.4,0.4)>

**Table 6:** P2TLN decision matrix.

	$\xi_{1}$	$\xi_{2}$	$\xi_{3}$
$A_{1}$	<(s_3_,−0.45), (0.6894,0.4826)>	<(s_3_,0.47), (0.5973,0.4811)>	<(s_2_,0.27), (0.4784,0.2461)>
$A_{2}$	<(s_2_,−0.01), (0.3719,0.3632)>	<(s_2_,0.16), (0.5992,0.437)>	<(s_3_,0.4), (0.6314,0.8053)>
$A_{3}$	<(s_2_,0.07), (0.6359,0.5056)>	<(s_4_,0.11), (0.5512,0.2305)>	<(s_0_,0.16), (0.3129,0.5006)>
$A_{4}$	<(s_4_,−0.27), (0.4219,0.4014)>	<(s_1_,0.38), (0.5885,0.4284)>	<(s_5_,−0.29), (0.6084,0.3559)>
$A_{5}$	<(s_4_,0.21), (0.7206,0.3103)>	<(s_4_,−0.37), (0.7674,0.3603)>	<(s_2_,−0.3), (0.4103,0.4831)>
	$\xi_{4}$	$\xi_{5}$	-
$A_{1}$	<(s_4_,−0.24), (0.5971,0.4989)>	<(s_2_,0.23), (0.6353,0.4391)>	-
$A_{2}$	<(s_3_,−0.42), (0.6492,0.3323)>	<(s_5_,0.11), (0.4945,0.4547)>	-
$A_{3}$	<(s_1_,0.23), (0.6412,0.3049)>	<(s_2_,−0.05), (0.6496,0.268)>	-
$A_{4}$	<(s_3_,0.24), (0.54,0.4249)>	<(s_4_,−0.05), (0.6965,0.273)>	-
$A_{5}$	<(s_4_,−0.3), (0.7065,0.3846)>	<(s_3_,0.26), (0.7848,0.4012)>	-

**Table 7:** Weighted normalized performance values of alternatives.

	$\xi_{1}$	$\xi_{2}$	$\xi_{3}$	$\xi_{4}$	$\xi_{5}$
$A_{1}$	1.0000	0.3384	0.0946	0.7434	0.2635
$A_{2}$	0.3384	1.0000	−0.8769	−0.0305	−0.6384
$A_{3}$	0.0946	−0.8769	1.0000	0.3326	0.6388
$A_{4}$	0.7434	−0.0305	0.3326	1.0000	0.3903
$A_{5}$	0.2635	−0.6384	0.6388	0.3903	1.0000

**Table 8:** Standard deviation of each attribute.

	$\xi_{1}$	$\xi_{2}$	$\xi_{3}$	$\xi_{4}$	$\xi_{5}$
$std_{j}$	0.7864	0.8078	1.0517	0.6239	0.7115

**Table 9:** Weights of attributes.

	$\xi_{1}$	$\xi_{2}$	$\xi_{3}$	$\xi_{4}$	$\xi_{5}$
$\omega_{j}$	0.1417	0.2961	0.2821	0.1126	0.1676

**Table 10:** Relative weight of the alternative $A_{1}$ to $A_{k}$.

	$A_{1}$	$A_{2}$	$A_{3}$	$A_{4}$	$A_{5}$
$A_{1}$	0.0000	0.0000	0.0000	0.0000	0.0000
$A_{2}$	1.0000	0.9732	0.9781	1.0000	0.9668
$A_{3}$	1.0000	1.0000	0.9781	1.0000	1.0000
$A_{4}$	0.9491	0.9732	1.0000	0.9253	0.9668
$A_{5}$	0.9491	1.0000	0.9781	0.9253	0.9668

**Table 11:** Transformed probability of the alternative $A_{2}$ to $A_{k}$.

	$A_{1}$	$A_{2}$	$A_{3}$	$A_{4}$	$A_{5}$
$A_{1}$	0.6448	1.0000	0.9712	0.5622	0.7366
$A_{2}$	0.0000	0.0000	0.0000	0.0000	0.0000
$A_{3}$	0.6448	1.0000	0.9499	0.6076	0.7366
$A_{4}$	0.6625	1.0000	0.9979	0.5777	0.7318
$A_{5}$	0.6448	1.0000	0.9499	0.5622	0.7366

**Table 12:** Transformed probability of the alternative $A_{3}$ to $A_{k}$.

	$A_{1}$	$A_{2}$	$A_{3}$	$A_{4}$	$A_{5}$
$A_{1}$	0.6625	1.0000	0.9979	0.5777	0.7318
$A_{2}$	0.6980	1.0000	0.9979	0.5777	0.7318
$A_{3}$	0.0000	0.0000	0.0000	0.0000	0.0000
$A_{4}$	0.6625	1.0000	0.9979	0.5777	0.7318
$A_{5}$	0.6448	1.0000	0.9712	0.5622	0.7122

**Table 13:** Transformed probability of the alternative $A_{4}$ to $A_{k}$.

	$A_{1}$	$A_{2}$	$A_{3}$	$A_{4}$	$A_{5}$
$A_{1}$	0.6793	1.0000	0.9499	0.5622	0.7366
$A_{2}$	0.6793	1.0000	0.9499	0.6076	0.7366
$A_{3}$	0.6793	1.0000	0.9499	0.6076	0.7366
$A_{4}$	0.0000	0.0000	0.0000	0.0000	0.0000
$A_{5}$	0.6448	1.0000	0.9499	0.5622	0.7366

**Table 14:** Transformed probability of the alternative $A_{5}$ to $A_{k}$.

	$A_{1}$	$A_{2}$	$A_{3}$	$A_{4}$	$A_{5}$
$A_{1}$	0.6980	1.0000	0.9979	0.6243	0.7569
$A_{2}$	0.6980	1.0000	0.9979	0.6243	0.7318
$A_{3}$	0.6980	1.0000	0.9760	0.6243	0.7569
$A_{4}$	0.6980	1.0000	0.9979	0.6243	0.7318
$A_{5}$	0.0000	0.0000	0.0000	0.0000	0.0000

**Table 15:** Relative prospect dominance $\varphi_{1}$.

	$A_{1}$	$A_{2}$	$A_{3}$	$A_{4}$	$A_{5}$
$A_{1}$	0.0000	0.0000	0.0000	0.0000	0.0000
$A_{2}$	0.0261	0.0295	0.0030	0.0184	−2.9824
$A_{3}$	0.0183	−1.5150	0.0497	0.0513	0.0021
$A_{4}$	−0.8475	0.0472	−3.3896	0.0131	−3.1835
$A_{5}$	−3.1971	−1.6285	0.0240	−1.1223	−2.3616

**Table 16:** Relative prospect dominance $\varphi_{2}$.

	$A_{1}$	$A_{2}$	$A_{3}$	$A_{4}$	$A_{5}$
$A_{1}$	−1.7549	−1.3146	−0.1357	−1.4172	0.0490
$A_{2}$	0.0000	0.0000	0.0000	0.0000	0.0000
$A_{3}$	−0.6511	−2.3206	0.0588	0.0285	0.0498
$A_{4}$	−2.5273	0.0262	−2.8786	−0.4534	−0.4316
$A_{5}$	−5.1534	−2.4138	0.0265	−2.6703	0.0128

**Table 17:** Relative prospect dominance $\varphi_{3}$.

	$A_{1}$	$A_{2}$	$A_{3}$	$A_{4}$	$A_{5}$
$A_{1}$	−1.2306	0.0341	−2.2640	−3.9477	−0.1277
$A_{2}$	0.0083	0.0654	−2.2032	−2.8824	−3.2821
$A_{3}$	0.0000	0.0000	0.0000	0.0000	0.0000
$A_{4}$	−2.0551	0.0859	−4.6643	−3.1651	−3.5396
$A_{5}$	−4.7206	−0.1917	−1.4068	−5.1028	−2.6629

**Table 18:** Relative prospect dominance $\varphi_{4}$.

	$A_{1}$	$A_{2}$	$A_{3}$	$A_{4}$	$A_{5}$
$A_{1}$	0.0128	−2.0654	0.0757	−1.0676	0.0533
$A_{2}$	0.0321	−0.9313	0.0769	0.0045	0.0066
$A_{3}$	0.0261	−3.0501	0.1246	0.0313	0.0538
$A_{4}$	0.0000	0.0000	0.0000	0.0000	0.0000
$A_{5}$	−3.0504	−3.1046	0.0982	−2.3516	0.0180

**Table 19:** Relative prospect dominance $\varphi_{5}$.

	$A_{1}$	$A_{2}$	$A_{3}$	$A_{4}$	$A_{5}$
$A_{1}$	0.0479	0.0368	−1.0888	0.0147	0.0391
$A_{2}$	0.0653	0.0680	−0.9909	0.0264	−0.8396
$A_{3}$	0.0599	0.0054	0.0376	0.0505	0.0404
$A_{4}$	0.0387	0.0875	−3.6781	0.0233	−1.1858
$A_{5}$	0.0000	0.0000	0.0000	0.0000	0.0000

**Table 20:** Value of $\phi \left(A_{i},A_{k}\right)$.

	$A_{1}$	$A_{2}$	$A_{3}$	$A_{4}$	$A_{5}$
$\phi \left(A_{i}\right)$	0.6455	0.5367	0.0000	0.7822	1.0000

**Table 21:** Aggregating result of the P2TLWA/P2TLWG operator.

	P2TLWA	P2TLWG
$A_{1}$	<(s_3_,−0.174), (0.592,0.394)>	<(s_3_,−0.2386), (0.5785,0.427)>
$A_{2}$	<(s_3_,0.0273), (0.577,0.4937)>	<(s_3_,−0.1402), (0.5553,0.591)>
$A_{3}$	<(s_2_,0.0205), (0.5506,0.3393)>	<(s_1_,0.1501), (0.5013,0.3876)>
$A_{4}$	<(s_3_,0.2924), (0.5934,0.3732)>	<(s_3_,−0.0507), (0.5773,0.3829)>
$A_{5}$	<(s_3_,0.1137), (0.694,0.393)>	<(s_3_,−0.0542), (0.6339,0.4037)>

**Table 22:** Results of alternatives.

Method Name		Scores				Order
	$A_{1}$	$A_{2}$	$A_{3}$	$A_{4}$	$A_{5}$	
P2TLWA	1.6890	1.6486	1.2001	1.9966	2.0662	$A_{5}>A_{4}>A_{1}>A_{2}>A_{3}$
P2TLWG	1.5910	1.3715	0.6331	1.7499	1.8247	$A_{5}>A_{4}>A_{1}>A_{2}>A_{3}$

**Table 23:** P2TL-TODIM calculation steps.

Steps	Calculation Results
Step 1. Identify the P2TLN decision matrix.	See Table 6
Step 2. Calculate the relative weight.	$w_{j}=\left(0.4786,1.0000,0.9528,\;0.3803,\;0.5659\;\right)$
Step 3. Calculate the dominance degree.	Because the calculation is too long, it is omitted here.
Step 4. Calculate the overall dominance degree	$\vartheta_{i}=\left(-1.8537,-2.8615,-5.5284,-0.1020,1.2534\;\right)$
Step 5. Derive the overall value.	$\xi_{i}=\left(0.5418,0.3932,0.0000,0.8001,1.0000\;\right)$
Step 6. Determine the ranking.	$A_{5}>A_{4}>A_{1}>A_{2}>A_{3}$

## References

[1] Drucker P. (1984). Converting social problem sinto business opportunities: The new meaning of corporate social responsibility. Calif. Manag. Rev..

[2] Najam A. (2000). The Four-C’s of third secto-government relations: Cooperation, confrontation, complementarity, and co-operation. Nonprofit Manag. Leadersh..

[3] Hastak M., Maris M.B., Morris L.A. (2000). The role of consumer surveys in public pollicy decision making. J. Public Policy Market..

[4] Revilla E., Sarkis J., Modrego A. (2003). Evaluating performance of public-private research collaborations: A DEA analysis. J. Oper. Res. Soc..

[5] Berrios R. (2006). Government contracts and contractor behavior. J. Bus. Eth..

[6] Ancarani A. (2009). Supplier evaluation in local public; Services: Application of a model of value for customer. J. Purch. Supply Manag..

[7] Grizzle G.A. (1999). Measuring State and Local Government Performance: Issues to Resolve Before Implementing a Performance Measuring System.

[8] Kearney R., Berman E. (1999). Public Sector Performance: Management Motivation and Measurement.

[9] Ammons D.N. (2001). Municipal Benchmarks: Assessing Local Performance and Establishing Community Standards.

[10] Waters H.R., Morlock L.L., Hatt L. (2004). Quality-based purchasing in health care. Int. J. Health Plan. Manag..

[11] Huang X., Mahieu R.J. (2012). Performance persistence of Dutch pension funds. Economist.

[12] Gomes L.F.A.M., Lima M.M.P.P. (1991). TODIM: Basic and application to multicriteria ranking of projects with environmental impacts. Found. Comput. Decis. Sci..

[13] Tian X.L., Xu Z.S., Gu J. (2019). An extended TODIM based on cumulative prospect theory and its application in venture capital. Informatica.

[14] Atanassov K.T. (1986). Intuitionistic fuzzy sets. Fuzzy Sets Syst..

[15] Zadeh L.A. (1965). Fuzzy sets. Inf. Control.

[16] Yager R.R. (2014). Pythagorean membership grades in multicriteria decision making. IEEE Trans. Fuzzy Syst..

[17] Wei G.W., Lu M., Alsaadi F.E., Hayat T., Alsaedi A. (2017). Pythagorean 2-tuple linguistic aggregation operators in multiple attribute decision making. J. Intell. Fuzzy Syst..

[18] Dick S., Yager R.R., Yazdanbakhsh O. (2016). On Pythagorean and Complex Fuzzy Set Operations. IEEE Trans. Fuzzy Syst..

[19] Herrera F., Martinez L. (2001). The 2-tuple linguistic computational model. Advantages of its linguistic description, accuracy and consistency. Int. J. Uncertain. Fuzziness Knowl. Based Syst..

[20] Huang Y.H., Wei G.W. (2018). TODIM method for Pythagorean 2-tuple linguistic multiple attribute decision making. J. Intell. Fuzzy Syst..

[21] Tang X.Y., Wei G.W. (2018). Models for green supplier selection in green supply chain management with Pythagorean 2-tuple linguistic information. IEEE Access.

[22] He T.T., Wei G.W., Lu J.P., Wei C., Lin R. (2019). Pythagorean 2-tuple linguistic VIKOR method for evaluating human factors in construction project management. Mathematics.

[23] He T.T., Wei G.W., Lu J.P., Wei C., Lin R. (2019). Pythagorean 2-tuple linguistic taxonomy method for supplier selection in medical instrument industries. Int. J. Environ. Res. Public Health.

[24] Atanassov K.T. (1989). More on intuitionistic fuzzy-sets. Fuzzy Sets Syst..

[25] Zhou W., Xu Z.S. (2018). Extended intuitionistic fuzzy sets based on the hesitant fuzzy membership and their application in decision making with risk preference. Int. J. Intell. Syst..

[26] Tang X.Y., Wei G.W. (2019). Dual hesitant Pythagorean fuzzy Bonferroni mean operators in multi-attribute decision making. Arch. Control Sci..

[27] Wu L.P., Gao H., Wei C. (2019). VIKOR method for financing risk assessment of rural tourism projects under interval-valued intuitionistic fuzzy environment. J. Intell. Fuzzy Syst..

[28] Lu J.P., Wei C. (2019). TODIM method for performance appraisal on social-integration-based rural reconstruction with interval-valued intuitionistic fuzzy information. J. Intell. Fuzzy Syst..

[29] Wang R. (2019). Research on the application of the financial investment risk appraisal models with some interval number muirhead mean operators. J. Intell. Fuzzy Syst..

[30] Wei G.W. (2019). 2-tuple intuitionistic fuzzy linguistic aggregation operators in multiple attribute decision making. Iran. J. Fuzzy Syst..

[31] Hadjitodorov S.T. (2001). An intuitionistic fuzzy version of the nearest prototype classification method, based on a moving-of-pattern procedure. Int. J. Gen. Syst..

[32] Hung W.L. (2002). Partial correlation coefficients of intuitionistic fuzzy sets. Int. J. Uncertain. Fuzziness Knowl. Based Syst..

[33] Xu Z.S., Yager R.R. (2006). Some geometric aggregation operators based on intuitionistic fuzzy sets. Int. J. Gen. Syst..

[34] Zhou J.M., Balezentis T., Streimikiene D. (2019). Normalized weighted bonferroni harmonic mean-based intuitionistic fuzzy operators and their application to the sustainable selection of search and rescue robots. Symmetry.

[35] Cavallaro F., Zavadskas E.K., Streimikiene D., Mardani A. (2019). Assessment of concentrated solar power (CSP) technologies based on a modified intuitionistic fuzzy topsis and trigonometric entropy weights. Technol. Forecast. Soc. Chang..

[36] Garg H. (2019). Generalized intuitionistic fuzzy entropy-based approach for solving multi-attribute decision-making problems with unknown attribute weights. Proc. Natl. Acad. Sci. India Sect. A Phys. Sci..

[37] Liu P.D., Li D.F. (2017). Some muirhead mean operators for intuitionistic fuzzy numbers and their applications to group decision making. PLoS ONE.

[38] Wu L.P., Wei G.W., Wu J., Wei C. (2020). Some Interval-valued intuitionistic fuzzy dombi heronian mean operators and their application for evaluating the ecological value of forest ecological tourism demonstration areas. Int. J. Environ. Res. Public Health.

[39] Mohammadi S.E., Makui A. (2017). Multi-attribute group decision making approach based on interval-valued intuitionistic fuzzy sets and evidential reasoning methodology. Soft Comput..

[40] Wan S.P., Wang F., Xu G.L., Dong J.Y., Tang J. (2017). An intuitionistic fuzzy programming method for group decision making with interval-valued fuzzy preference relations. Fuzzy Optim. Decis. Mak..

[41] Garg H., Kumar K. (2018). An advanced study on the similarity measures of intuitionistic fuzzy sets based on the set pair analysis theory and their application in decision making. Soft Comput..

[42] Kaur G., Garg H. (2018). Multi-attribute decision-making based on bonferroni mean operators under cubic intuitionistic fuzzy set environment. Entropy.

[43] Wu L.P., Wang J., Gao H. (2019). Models for competiveness evaluation of tourist destination with some interval-valued intuitionistic fuzzy Hamy mean operators. J. Intell. Fuzzy Syst..

[44] Chen S.M., Kuo L.W. (2018). Multiattribute decision making based on non-linear programming methodology with hyperbolic function and interval-valued intuitionistic fuzzy values. Inf. Sci..

[45] Chen S.M., Kuo L.W., Zou X.Y. (2018). Multiattribute decision making based on Shannon’s information entropy, non-linear programming methodology, and interval-valued intuitionistic fuzzy values. Inf. Sci..

[46] Garg H., Kumar K. (2019). Linguistic interval-valued atanassov intuitionistic fuzzy sets and their applications to group decision making problems. IEEE Trans. Fuzzy Syst..

[47] Arora R., Garg H. (2019). Group decision-making method based on prioritized linguistic intuitionistic fuzzy aggregation operators and its fundamental properties. Comput. Appl. Math..

[48] Zhang X.L., Xu Z.S. (2014). Extension of TOPSIS to multiple criteria decision making with Pythagorean fuzzy sets. Int. J. Intell. Syst..

[49] Liang D.C., Zhang Y.R.J., Xu Z.S., Darko A.P. (2018). Pythagorean fuzzy Bonferroni mean aggregation operator and its accelerative calculating algorithm with the multithreading. Int. J. Intell. Syst..

[50] Peng X.D., Yang Y. (2015). Some results for Pythagorean fuzzy sets. Int. J. Intell. Syst..

[51] Li Z.X., Lu M. (2019). Some novel similarity and distance and measures of Pythagorean fuzzy sets and their applications. J. Intell. Fuzzy Syst..

[52] Garg H. (2019). New logarithmic operational laws and their aggregation operators for Pythagorean fuzzy set and their applications. Int. J. Intell. Syst..

[53] Ren P.J., Xu Z.S., Gou X.J. (2016). Pythagorean fuzzy TODIM approach to multi-criteria decision making. Appl. Soft Comput..

[54] Zeng S.Z., Peng X.M., Balezentis T., Streimikiene D. (2019). Prioritization of low-carbon suppliers based on Pythagorean fuzzy group decision making with self-confidence level. Econ. Res. Ekon. Istraz..

[55] Gul M., Ak M.F., Guneri A.F. (2019). Pythagorean fuzzy VIKOR-based approach for safety risk assessment in mine industry. J. Saf. Res..

[56] Yu L.P., Zeng S.Z., Merigo J.M., Zhang C.H. (2019). A new distance measure based on the weighted induced method and its application to Pythagorean fuzzy multiple attribute group decision making. Int. J. Intell. Syst..

[57] Liang D.C., Zhang Y.R.J., Xu Z.S., Jamaldeen A. (2019). Pythagorean fuzzy VIKOR approaches based on TODIM for evaluating internet banking website quality of Ghanaian banking industry. Appl. Soft Comput..

[58] Zeb A., Khan M.S.A., Ibrar M. (2019). Approaches to multi-attribute decision making with risk preference under extended Pythagorean fuzzy environment. J. Intell. Fuzzy Syst..

[59] Gou X.J., Xu Z.S., Ren P.J. (2016). The properties of continuous Pythagorean fuzzy information. Int. J. Intell. Syst..

[60] Liang D.C., Xu Z.S., Darko A.P. (2017). Projection model for fusing the information of Pythagorean fuzzy multicriteria group decision making based on geometric bonferroni mean. Int. J. Intell. Syst..

[61] Chen T.Y. (2018). A novel promethee-based outranking approach for multiple criteria decision analysis with Pythagorean fuzzy information. IEEE Access.

[62] Thao N.X., Smarandache F. (2019). A new fuzzy entropy on Pythagorean fuzzy sets. J. Intell. Fuzzy Syst..

[63] Chen T.Y. (2018). A mixed-choice-strategy-based consensus ranking method for multiple criteria decision analysis involving Pythagorean fuzzy information. IEEE Access.

[64] Teng F., Liu Z.M., Liu P.D. (2018). Some power Maclaurin symmetric mean aggregation operators based on Pythagorean fuzzy linguistic numbers and their application to group decision making. Int. J. Intell. Syst..

[65] Ma Z.M., Xu Z.S. (2016). Symmetric Pythagorean fuzzy weighted geometric/averaging operators and their application in multicriteria decision-making problems. Int. J. Intell. Syst..

[66] Peng X.D., Yang Y. (2016). Pythagorean fuzzy choquet integral based MABAC method for multiple attribute group decision making. Int. J. Intell. Syst..

[67] Garg H. (2018). Generalised Pythagorean fuzzy geometric interactive aggregation operators using Einstein operations and their application to decision making. J. Exp. Theor. Artif. Intell..

[68] Li Z.X., Wei G.W., Lu M. (2018). Pythagorean fuzzy hamy mean operators in multiple attribute group decision making and their application to supplier selection. Symmetry.

[69] Chen T.Y. (2018). An effective correlation-based compromise approach for multiple criteria decision analysis with Pythagorean fuzzy information. J. Intell. Fuzzy Syst..

[70] Zhang X.L. (2016). Multicriteria Pythagorean fuzzy decision analysis: A hierarchical QUALIFLEX approach with the closeness index-based ranking methods. Inf. Sci..

[71] Garg H. (2017). Confidence levels based Pythagorean fuzzy aggregation operators and its application to decision-making process. Comput. Math. Organ. Theory.

[72] Wei G.W., Garg H., Gao H., Wei C. (2018). Interval-valued Pythagorean fuzzy maclaurin symmetric mean operators in multiple attribute decision making. IEEE Access.

[73] Tang X.Y., Wei G.W., Gao H. (2019). Pythagorean fuzzy Muirhead mean operators in multiple attribute decision making for evaluating of emerging technology commercialization. Econ. Res. Ekon. Istraz..

[74] Tang X.Y., Wei G.W., Gao H. (2019). Models for multiple attribute decision making with interval-valued Pythagorean fuzzy muirhead mean operators and their application to green suppliers selection. Informatica.

[75] Geng Y.S., Liu P.D., Teng F., Liu Z.M. (2017). Pythagorean fuzzy uncertain linguistic TODIM method and their application to multiple criteria group decision making. J. Intell. Fuzzy Syst..

[76] Chen T.Y. (2018). Remoteness index-based Pythagorean fuzzy VIKOR methods with a generalized distance measure for multiple criteria decision analysis. Inf. Fus..

[77] Deng X.M., Wang J., Wei G.W. (2019). Some 2-tuple linguistic Pythagorean Heronian mean operators and their application to multiple attribute decision-making. J. Exp. Theor. Artif. Intell..

[78] Garg H. (2018). Linguistic Pythagorean fuzzy sets and its applications in multiattribute decision-making process. Int. J. Intell. Syst..

[79] Wang J., Wei G.W., Gao H. (2018). Approaches to multiple attribute decision making with interval-valued 2-tuple linguistic Pythagorean fuzzy information. Mathematics.

[80] Diakoulaki D., Mavrotas G., Papayannakis L. (1995). Determining objective weights in multiple criteria problems: The critic method. Comput. Oper. Res..

[81] Lu J.P., Tang X.Y., Wei G.W., Wei C., Wei Y. (2019). Bidirectional project method for dual hesitant Pythagorean fuzzy multiple attribute decision-making and their application to performance assessment of new rural construction. Int. J. Intell. Syst..

[82] Lu J.P., Wei C., Wu J., Wei G.W. (2019). TOPSIS method for probabilistic linguistic MAGDM with entropy weight and its application to supplier selection of new agricultural machinery products. Entropy.

[83] Wang P., Wang J., Wei G.W., Wei C., Wei Y. (2019). The multi-attributive border approximation area comparison (MABAC) for multiple attribute group decision making under 2-tuple linguistic neutrosophic environment. Informatica.

[84] Wang P., Wei G.W., Wang J., Lin R., Wei Y. (2019). Dual hesitant q-rung orthopair fuzzy hamacher aggregation operators and their applications in scheme selection of construction project. Symmetry.

[85] Wei G.W., Wang R., Wang J., Wei C., Zhang Y. (2019). Methods for Evaluating the Technological Innovation Capability for the High-Tech Enterprises With Generalized Interval Neutrosophic Number Bonferroni Mean Operators. IEEE Access.

[86] Zavadskas E.K., Antucheviciene J., Chatterjee P. (2019). Multiple-Criteria Decision-Making (MCDM) Techniques for Business Processes Information Management. Information.

[87] Wang J., Gao H., Lu M. (2019). Approaches to strategic supplier selection under interval neutrosophic environment. J. Intell. Fuzzy Syst..

[88] Zavadskas E.K., Bausys R., Mazonaviciute I. (2019). Safety evaluation methodology of urban public parks by multi-criteria decision making. Landsc. Urban Plan..

[89] He Y., Lei F., Wei G.W., Wang R., Wu J., Wei C. (2019). EDAS method for multiple attribute group decision making with probabilistic uncertain linguistic information and its application to green supplier selection. Int. J. Comput. Intell. Syst..

[90] Lei F., Wei G.W., Lu J.P., Wei C., Wu J. (2019). GRA method for probabilistic linguistic multiple attribute group decision making with incomplete weight information and its application to waste incineration plants location problem. Int. J. Comput. Intell. Syst..

[91] Wei G.W., Wei C., Wu J., Wang H.J. (2019). Supplier selection of medical consumption products with a probabilistic linguistic MABAC method. Int. J. Environ. Res. Public Health.

[92] Gao H., Ran L.G., Wei G.W., Wei C., Wu J. (2020). VIKOR method for MAGDM based on q-rung interval-valued orthopair fuzzy information and its application to supplier selection of medical consumption products. Int. J. Environ. Res. Public Health.

[93] Li Z.X., Wei G.W., Wang R., Wu J., Wei C., Wei Y. (2020). EDAS method for multiple attribute group decision making under q-rung orthopair fuzzy environment. Technol. Econ. Dev. Econ..

[94] Angiulli G., Versaci M. (2002). A Neuro-Fuzzy Network for the Design of Circular and Triangular Equilateral Microstrip Antennas. Int. J. Infrared Millim. Waves.

[95] Cacciola M., Pellicanò D., Megali G., Lay-Ekuakille A., Versaci M., Morabito F.C. Aspects about air pollution prediction on urban environment. Proceedings of the 4th IMEKO TC19 Symposium on Environmental Instrumentation and Measurements.

